# Cesarean Section and Right Femur Fracture: A Rare but Possible Complication for Breech Presentation

**DOI:** 10.1155/2013/613709

**Published:** 2013-03-06

**Authors:** Giampiero Capobianco, Giuseppe Virdis, Pietro Lisai, Claudio Cherchi, Ornella Biasetti, Francesco Dessole, Giovanni Battista Meloni

**Affiliations:** ^1^Department of Surgical, Microsurgical and Medical Sciences, Gynecologic and Obstetric Clinic, University of Sassari, 07100 Sassari, Italy; ^2^Department of Surgical, Microsurgical and Medical Sciences, Institute of Orthopedics, University of Sassari, 07100 Sassari, Italy; ^3^Neonatology Intensive Care Unit, University of Sassari, 07100 Sassari, Italy; ^4^Department of Surgical, Microsurgical and Medical Sciences, Institute of Radiology, University of Sassari, 07100 Sassari, Italy

## Abstract

*Background*. The breech extraction of the fetus through the vagina has a greater risk of hip fracture compared with the extraction by abdominal route. *Case*. A 2390 g female infant was delivered at 39 weeks by elective cesarean section for breech presentation. The newborn sustained a fracture of the right femur. A simple immobilization of the limb in extension led to a complete healing of the fracture without sequelae. *Conclusion*. Caesarean delivery reduces the risk of causing a traumatic injury of the newborn compared to vaginal delivery, especially with breech presentation but does not eliminate this possible accidental complication.

## 1. Introduction

The vaginal breech birth, even if rarely, can lead to traumatic outcome as a fracture of the femur [1]. For cesarean section with breech presentation, such cases are even if more rare, but still possible, as reported in the literature [2–8]. The multicenter, randomized study of Hannah et al. [9] showed that the fracture of long bones occurred in 0.1% of cases during caesarean section and 0.5% for vaginal delivery.

Planning caesarean section reduces the risk of fracture of long bones but does not eliminate the possibility [10–13]. 

We present a case of right femur fracture that occurred in the course of cesarean section performed because of breech presentation.

## 2. The Case 

The case concerns a 31-year-old Caucasian patient who underwent a cesarean section for breech presentation at 39 weeks of gestation with a history of hysterotomy because of multiple myomas in 2005. 

The patient underwent a vaginal delivery in 2003. The patient was subjected to epidural anesthesia to obtain an adequate analgesia and muscle relaxation. The incision was performed on the lower uterine segment, with adequate width. The fetus showed a frank transverse situation, the iliac crest of the podex fetal found to be below the incision. The operator has engaged the left thigh and performed a pull to extract the fetus as usual in a breech extraction during cesarean section. The breech was engaged without difficulty, and the fetus was extracted directly by the lower limbs. During the extraction, we did not hear any suspicious sounds (crack) [12]. A 2390 g female infant was delivered with Apgar scores of 9 and 10 at 1 and 5 min, respectively.

At the time of extraction, we did not pay particular attention to the right thigh of the newborn. The baby was subjected to careful clinical examination, and routine laboratory tests showed no abnormalities. 

**Figure 1:**
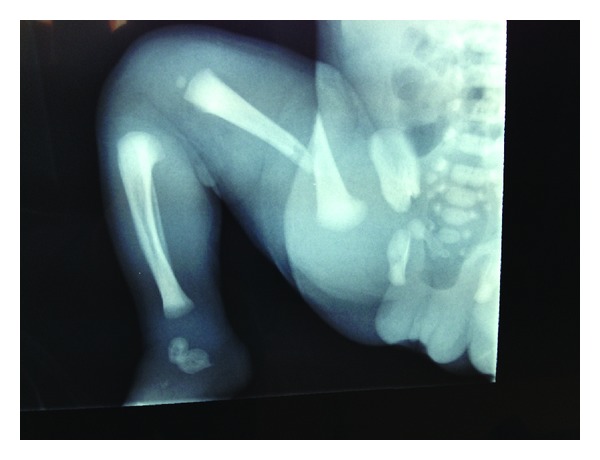
Rx showing right femur fracture.

On the day after birth, the neonate remained irritable and was disinterested in feeding; the right thigh had a step on palpation and reduced mobility. The clinical signs led the neonatology team to perform an X-ray of the legs of the newborn. The examination revealed a fracture of the right femur in which the proximal segment displaced anteriorly compared to the middle and distal segment (Figure 1). The bone structure and mineralization were visibly normal; there was no indication of any other fracture/bone deformities or anomalies osteo-articular (blue sclera, osteogenesis imperfecta, or hypotonia). In particular, Welding-Hofmann disease, the pathology that promotes bone atrophy and facilitates pathologic fractures of long bones, was excluded.

**Figure 2:**
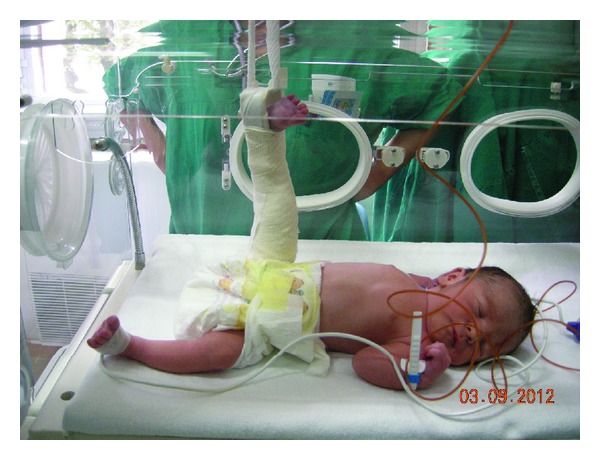
Immobilization of the right leg in extension.

The newborn was treated with immobilization in extension (Figure 2). 

**Figure 3:**
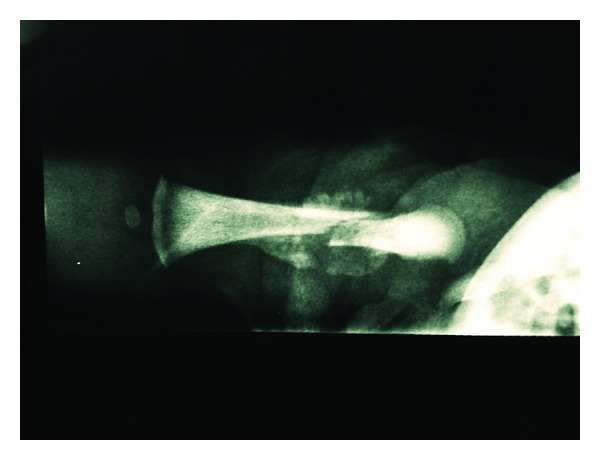
Rx showing the healing of the fracture.

On the tenth day after birth, the radiogram control showed the formation of a callus at the level of the margins of fracture. At 20 days a further control radiogram showed progression of the callus (Figure 3). It was decided to remove the immobilization 23 days after birth; the child was able to move his right leg actively in all planes of space. At the 75th day after birth, the fracture was found to be fully welded. 

At 20 weeks after the birth, both lower limbs showed a proper mobility with no dysmetria.

## 3. Discussion

We have already demonstrated that cesarean section induced 3.12% accidental fetal lacerations [14]. In the medical literature, there are only nine articles reporting cases of hip fracture during cesarean section [1–5, 7, 10, 12, 13]. This possibility, as is reported in the literature, is extremely rare. Some conditions that can be regarded as favoring the above complications are mainly represented by twin pregnancy, inadequate uterine relaxation, the presence of myomas, inadequate incision in the lower uterine segment, and also the presentation of breech well engaged in the pelvis. The current lack of available data and the extreme rarity of this complication do not detect an adequate and precise maneuvering to avoid fracture of the femur. An appropriate set of rules and procedures may prevent the occurrence of such an incident. These rules are represented by an adequate analgesia, extraction by exercising a delicate traction, by performing an uterine incision that is sufficiently wide to allow a smooth extraction. In this regard, it is recommended an extension of the uterine incision rather than continue to exert traction difficult and/or dangerous. The occurrence of characteristic sound (crack) [12] may be regarded as an important sign to put on suspicion of breaking the femur of the newborn during extraction. This finding should prompt when warned to carry out further investigations such as X-rays of the lower limbs of the newborn, all in accordance with the neonatologists. These recommendations represent the process more correctly for early detection of this complication, in order to prepare an early treatment of the condition. In our case, there were no predisposing conditions. The extraction was apparently simple, without particular or excessively energetic tractions. The occurrence of fracture of the femur in the neonatal was a real surprise. This case increases the awareness of the clinical complications in the course of cesarean section.

## 4. Conclusion

Cesarean delivery reduces the risk of causing a traumatic injury of the newborn compared to vaginal delivery, especially with breech presentation, but does not eliminate this possible accidental complication [15].
